# Sickness absence among migrant and non-migrant care workers in Finland: A register-based follow-up study

**DOI:** 10.1177/14034948231168434

**Published:** 2023-04-22

**Authors:** Antero Olakivi, Anne Kouvonen, Aki Koskinen, Laura Kemppainen, Lauri Kokkinen, Ari Väänänen

**Affiliations:** 1 Faculty of Social Sciences, University of Helsinki, Finland; 2 Centre for Public Health, Queen’s University Belfast, UK; 3 Finnish Institute of Occupational Health, Finland; 4 Faculty of Social Sciences, Tampere University, Finland

**Keywords:** Sickness absence, migrants, group comparison, health care, social care, care work, follow-up, register study, occupational health, presenteeism

## Abstract

**Aims:**

This study aimed to compare the sickness absence (SA; over 10 days) rates of migrant and non-migrant care workers in Finland.

**Methods:**

Two cohorts were randomly sampled from nationwide registers and analysed together in a three-year follow-up design (2011–2013, 2014–2016). The pooled data consisted of 78,476 care workers, of whom 5% had a migrant background. Statistical methods included cross-tabulations and Poisson regression modelling.

**Results:**

Thirty-five percent of the Finnish-born care workers had at least one SA during the follow-up. Care workers from the post-2004 EU countries (30%, at least one SA), Russia, the Former Soviet Union and the Balkan states (25%) and the Global South and East (21%) had fewer episodes of SA than the Finnish-born care workers. The two latter groups also had lower SA rates after we controlled for occupation, gender, age, income and region of residence. Care workers from Western Europe and the Global North (36%) had higher SA rates than the Finnish-born care workers.

**Conclusions:**

**The following explanations were discussed: population-level health differences – migrants from lower-income non-EU countries are generally healthier than the Finnish-born population (due to, e.g., the ‘healthy migrant effect’); discrimination in recruitment and employment – migrants from lower-income non-EU countries need to be healthier than Finnish-born jobseekers to gain employment (in the care sector or more broadly); and sickness presenteeism – migrants from lower-income non-EU countries underuse their right to sickness allowance (due to, e.g., job insecurity). It is likely that these mechanisms affect migrants differently depending on, for example, their countries of origin and social status in Finland.**

## Introduction

In Finland, as in other affluent countries, the proportion of migrants working in the care sector is growing rapidly, especially in urban areas [1–5]. In public discourse, this trend is typically presented as a response to shortages in care labour, shortages caused by aging populations and the persistent under-resourcing of paid care work [1–5]. In the Nordic countries (and elsewhere), significant evidence shows that migrant care workers, especially migrants from less affluent countries, receive lower-level positions and poorer treatment (e.g. more discrimination) than non-migrant care workers [1–5]. This study is the first to extend this research to comparing the sickness absence (SA) rates of migrant and non-migrant care workers in a design that uses nationwide registers. It focuses on paid care work in Finland, as there is a need for research on care workers’ occupational health, and the employment of migrants in the formal care sector is a specific political target (in Finland and other countries) [1–6].

According to previous research, care workers are vulnerable to various occupational hazards that are known predictors of SA [7,8], including low job control and mental and physical overload [9]. Migrants are overrepresented in the most burdensome and least autonomous jobs in the formal care sector [1–5]. A migrant status can expose care workers to additional vulnerabilities, including discrimination, racism and a lack of support from co-workers and supervisors [1–5]. These hazards are known to predict SA in care occupations [8,10]. Therefore, one might expect migrant care workers to have higher SA rates than their non-migrant peers.

Other underlying predictors of SA may introduce further complications for analyses of SA and their interpretation. Research shows that the following factors can increase the risk of sickness presenteeism, that is, working in spite of a health problem: understaffing and recruitment difficulties [7,11], job insecurity [12,13] and a lack of support from supervisors or colleagues (due to fear of unequal distribution of tasks and resources at work if absent) [12,14,15]. Research also suggests that a migrant status can strengthen all these predictors of presenteeism in care work, including temporary contracts [1,4] and the likelihood of working in labour shortage and understaffing conditions [1–6]. Prior evidence indicates that migrant workers’ risk of presenteeism is generally higher than average [13] also in the care sector [16], which has high rates of sickness presenteeism also among non-migrant workers [7,9,11].

Finally, evidence suggests that although some groups of migrants in Finland are at a higher than average risk of ill health according to some indicators [17,18], most migrant groups have lower than average mortality rates [19,20] and use health and rehabilitation services less frequently [21–23]. In addition to lifestyle differences and the ‘healthy migrant effect’ [20,24], these observations seem to imply underuse of medical and rehabilitation services and access disadvantages, especially among migrants from lower-income and non-EU countries [21,22–25].

To summarise, it is possible that labour market and workplace conditions may increase the SA rates of migrant care workers, whereas sociocultural mechanisms and factors related to employment status may decrease SA rates among care workers with migrant backgrounds. In this nationwide register study, we used a three-year follow-up design to compare the SA rates of Finnish-born workers with those of migrants from different countries/regions in paid care work in Finland.

## Data and methods

### Study design and data sources

We obtained the data from a population database maintained by Statistics Finland and a national register maintained by the Social Insurance Institution of Finland (SII). In the population database, every resident, including every non-citizen migrant with a permanent, continuous or temporary residence permit, is registered under a personal identification number. From these data, we obtained a 33% random sample of the working-age population (18–64 years at baseline) in two cohorts (2011–2013 and 2014–2016). The cohorts were analysed together to ensure adequate numbers for the analysis. The total working-age population was not selected, as the SII only allows a 33% random sample in order to guarantee participants’ anonymity. From the population database, we obtained data on each participant’s occupation, gender, age, annual income, region of residence (in Finland) and country/region of birth. For annual income, we used the average annual income of each participant during the follow-up.

Finally, we linked the population data to sickness allowance records obtained from the SII. Regardless of their employment status, all permanent residents aged 16–67 years are entitled to daily allowances of medically certified sick leave if they are unable to work in their regular field of employment in Finland. This includes non-citizen migrants with a permanent or continuous residence permit, as well as, under specific conditions, those with a temporary residence permit. After the first 10 calendar days of sick leave, the SII provides compensation for sick leave for a maximum of one year. If the applicant is employed, the employer usually applies for the compensation. Medical certification is required for each absence, and the start and end dates are recorded in the SII register.

We followed both cohorts over a three-year period, beginning on 1 January 2011 or 2014. The follow-up ended on the day that the participant was granted sickness allowance, became unemployed (unemployment was censored annually if the participant had at least two months per year) or died, whichever came first. The participants who were assigned SA for any cause, which lasted a minimum of 11 consecutive days, were classified as cases.

Countries/regions of birth were categorised as follows: Western Europe and the Global North (including Sweden and the other EU15 countries, Australia, Canada, New Zealand and the USA), post-2004 EU countries (including Estonia, Latvia, Lithuania and Poland, but excluding Croatia and Serbia), Russia, the Former Soviet Union and the Balkan states (including Croatia and Serbia, Albania, Belarus, Former Yugoslavia, Ukraine, etc.) and the Global South and East (including Africa, Latin America, Asia, Oceania, etc.). All individuals born in Finland were categorised as the settled majority for the purpose of comparison.

At the start of each cohort, we categorised the participants according to the International Standard Classification of Occupations (ISCO) codes [26]. The ISCO is an International Labour Organization (ILO) classification structure for organising jobs into defined sets of groups according to the tasks and duties undertaken in each field of work. In the present study, we focused on ISCO-08 groups 5321 (health-care assistants), 5322 (home-based personal care workers) and 5329 (personal care workers in health services not classified elsewhere). The first group is mainly employed in institutional settings, such as hospitals, clinics and nursing homes, and the second group primarily works in private homes. The third group includes occupations such as dental assistants, sterilisation assistants, hospital orderlies, medical imaging assistants, pharmacy assistant and other occupations that are not directly involved in daily physical care.

### Participants

**Figure 1.**
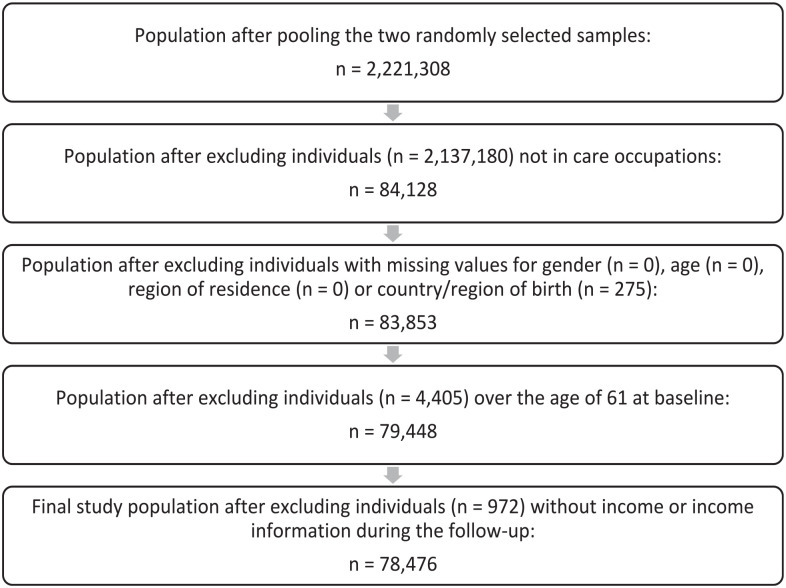
Inclusion and exclusion of study participants.

The analyses focused on participants in the above care occupations. Individuals were excluded if their gender, age, region of residence or country/region of birth information was missing. Individuals >61 years of age at baseline were excluded, as they would be likely to retire due to age during the follow-up, and this might cause bias in the analyses. Individuals with missing income information or no income during any year of the follow-up were excluded. The final data included 78,476 care workers (90% women, 5% migrants, mean age 40 years, median annual average income EUR 29,000). Figure 1 displays the inclusion process.

### Statistical analyses

We first used descriptive statistics (frequencies, percentages) to identify the socio-demographic differences between the migrants and the Finnish-born care workers. We then compared SA between the migrant groups and the settled majority using cross-tabulations and two Poisson regression models, the first adjusted for age, gender and cohort and the second adjusted for age, gender, cohort, income, the region of residence and occupation. The Finnish-born majority was designated as the reference group. In all models, survival time was used as an offset variable. IBM SPSS Statistics for Windows v28 (IBM Corp., Armonk, NY) was employed in the analyses. The results are reported as incidence rate ratios (RRs), including their 95% confidence intervals. Poisson modelling does not assume that the outcome phenomenon is normally distributed. It does assume, however, that the variance of the outcome variable among the study participants equals the mean. Our data are somewhat in line with this assumption, with the variance for SA being 0.23 and the mean 0.34.

The study adheres to the ethical standards set out by the 1975 Declaration of Helsinki and its later amendments.

## Results

Table I displays the main characteristics of the study population. The percentages of men in the migrant groups from Western Europe and the Global North and the Global South and East were higher than in the other groups. The migrants from the Global South and East were notably younger than the individuals in the other groups. Compared to the individuals in the other groups, the migrants from the Global South and East also encompassed a higher percentage of health care assistants. The migrants from Russia, the Former Soviet Union and the Balkan states comprised a higher percentage of personal care workers in health services not classified elsewhere. All the migrant groups had higher proportions of individuals in the lowest income tertile than the Finnish-born care workers. The Greater Helsinki (Uusimaa) area and the second cohort consisted of markedly high percentages of migrants, except for migrants from Western Europe and the Global North.

**Table I.** Care workers’ sociodemographic characteristics by country/region of origin and sickness absence (SA) by sociodemographic characteristics and country/region of origin.

Sociodemographic characteristics	Finland	Western Europe and the Global North	Post-2004 EU countries	Russia, the Former Soviet Union and the Balkan states	Global South and East	SA, at least one incidence
	*n* (%)	*n* (%)	*n* (%)	*n* (%)	*n* (%)	*n* (%)
Total	74,655	1021	504	1089	1207	27,065 (35)
Gender						
Women	67,729 (91)	837 (82)	477 (95)	1028 (94)	933 (77)	25,180 (36)
Men	6926 (9)	184 (18)	27 (5)	61 (6)	274 (23)	1885 (25)
Age (years)						
18–30	21,030 (28)	244 (24)	108 (21)	248 (23)	561 (47)	5162 (23)
31–40	13,539 (18)	372 (36)	134 (27)	242 (22)	356 (30)	4950 (34)
41–50	18,809 (25)	273 (27)	125 (25)	334 (31)	224 (19)	7590 (38)
51–60	21,277 (29)	132 (13)	137 (27)	265 (24)	66 (6)	9363 (43)
Occupation group						
ISCO-08 5321^a^	50,231 (67)	715 (70)	320 (64)	697 (64)	919 (76)	18,545 (35)
ISCO-08 5322^b^	15,209 (21)	210 (21)	117 (23)	205 (19)	207 (17)	5729 (36)
ISCO-08 5329^c^	9134 (12)	96 (9)	67 (13)	187 (17)	81 (7)	2791 (29)
Income tertile						
Lowest^d^	24,627 (33)	371 (36)	185 (37)	411 (38)	518 (43)	6512 (25)
Middle^e^	25,038 (34)	332 (33)	143 (28)	324 (30)	350 (29)	10,600 (41)
Highest^f^	24,990 (34)	318 (31)	176 (35)	354 (33)	339 (28)	9953 (38)
Region						
Greater Helsinki	15,498 (21)	199 (20)	316 (63)	436 (40)	733 (61)	5519 (32)
Other regions	59,157 (79)	822 (81)	188 (37)	653 (60)	474 (39)	21,546 (35)
Cohort						
2011–2013	37,470 (50)	494 (48)	206 (41)	496 (46)	507 (42)	13,736 (35)
2014–2016	37,185 (50)	527 (52)	298 (59)	593 (55)	700 (58)	13,329 (34)
SA, at least one incidence	26,033 (35)	365 (36)	152 (30)	268 (25)	247 (21)	

a Health-care assistants.

b Home-based personal care workers.

c Personal care workers in health services not classified elsewhere.

d Average annual income during the follow-up EUR 100–25,567 (median EUR 19,500).

e Average annual income during the follow-up EUR 25,600–32,167 (median EUR 29,200).

f Average annual income during the follow-up EUR 32,200–64,100 (median EUR 35,333).

Of the care workers, 35% (*n*=27,065) had at least one SA during the follow-up. SA was more prevalent among women and older participants and less prevalent among the personal care workers in health services not classified elsewhere, and among the individuals in the lowest income tertile and the Greater Helsinki region (Table I). Of the Finnish-born care workers, 35% had at least one SA during the follow-up. The care workers from the post-2004 EU countries (30% had at least one SA during the follow-up), Russia, the Former Soviet Union and the Balkan states (25%) and the Global South and East (21%) had lower SA rates than the Finnish-born care workers. The care workers from Western Europe and the Global North (36%) had marginally higher SA rates than the Finnish-born care workers.

Table II shows the Poisson regression models’ results. The first model shows that the care workers from the post-2004 EU countries, Russia, the Former Soviet Union and the Balkan states and the Global South and East had lower estimated SA rates than the Finnish-born care workers, also when age, gender and cohort were controlled for. These differences were slightly smaller when the second model was additionally adjusted for occupation, region and income. Indeed, the second model estimated that, at a confidence level of 95%, the care workers from the post-2004 EU countries did not necessarily have lower SA rates than the Finnish-born care workers. The care workers from Western Europe and the Global North had higher estimated SA rates than the Finnish-born care workers in both models.

**Table II.** Poisson regression rate ratios (RRs) and 95% confidence intervals (CIs) for sickness absence among care workers from different countries/regions of birth.

		Model 1	Model 2
	*n*	RR (95% CI)	RR (95% CI)
Finland	74,655		
Western Europe and the Global North	1021	1.20 (1.08–1.33)	1.19 (1.07–1.31)
Post-2004 EU countries	504	0.84 (0.72–0.99)	0.87 (0.74–1.02)
Russia, the Former Soviet Union and the Balkan states	1089	0.65 (0.58–0.74)	0.67 (0.59–0.76)
Global South and East	1207	0.66 (0.58–0.75)	0.67 (0.59–0.76)

Model 1: Adjusted for age (continuous), gender and cohort.

Model 2: Adjusted for age (continuous), gender, cohort, region, income (continuous) and occupation.

To test the robustness of the findings, we ran models 1 and 2 separately for (a) the two cohort samples, (b) 31- to 50-year-old participants, (c) women and (d) the two highest income tertiles. The results were highly consistent with those reported in Table II (see Supplemental Tables SI–SV). The confidence intervals were naturally larger, but all the models estimated lower SA rates for the care workers from Russia, the Former Soviet Union and the Balkan states and the Global South and East than for the Finnish-born care workers. Nine of the ten models estimated higher SA rates for the migrants from Western Europe and the Global North. Five of the ten models estimated lower SA rates for the post-2004 EU migrants than the Finnish-born care workers at a confidence level of 95%.

## Discussion

The proportion of migrants is growing in the Finnish care sector. In the present study, migrants from lower-income non-EU countries had lower SA rates than Finnish-born care workers. The same may apply to migrants from the post-2004 EU countries, but the risk of error in this conclusion is higher. When age and gender (among other factors) were controlled for, migrants from Western Europe and the Global North had higher estimated SA rates than Finnish-born care workers.

It is possible that migrants from the lower-income non-EU countries in particular have better overall health than Finnish-born care workers because of their lifestyle or the ‘healthy migrant effect’ [19,20,24]. According to population-based studies, health differences are notable between the settled majority population and migrants from the Global South and East [20]. Previous studies have also shown that non-EU migrants and migrants from lower-income countries in particular are at an increased risk of underusing health and rehabilitation services in Finland (due to, e.g., service system illiteracy) [21,22–25].

Selection bias may also have affected these results. Migrants face discrimination in the labour market [27], and in comparison to Finnish-born jobseekers, a migrant’s recruitment and employment may be more dependent on maintaining a healthy appearance and, for example, not having visible disabilities or not being obese. Migrant care workers may also be more educated because of occupational downgrading [1,28] and therefore have better health outcomes than their Finnish-born colleagues.

Sickness presenteeism is common in care work [7,9,11]. In previous studies, a lack of support from colleagues and supervisors [12,14,15] and job insecurity [12,13] have predicted presenteeism. According to previous research, migrants from the Global South and East in particular experience less support and more (racial) discrimination from their colleagues and supervisors than the non-migrant majority in Finland [1,29]. In Finland, non-EU migrants are less likely to have an EU certificate in care work and a permanent job in the health and social care sector [28]. Some non-EU care workers may have restricted access to social security (e.g. unemployment insurance) in Finland, and non-EU care workers’ residence permits may depend on their employment. All this make them more dependent on their employers, which can increase their risk of presenteeism.

Multiple factors may thus explain the differences in SA between Finnish-born care workers and migrants from lower-income non-EU countries. In a study by Carneiro et al. [16], migrant care workers had lower SA rates than their Danish-born peers, despite the migrants also having a poorer health status on average. However, research has shown that in settings with high efficiency demands (e.g. care work), the prevalence of sickness presenteeism also tends to be high, especially among workers who have relatively good overall health [30]. In summary, one has to be relatively healthy in order to be able to work while sick [13,14]. Therefore, better overall health and various presenteeism mechanisms may reinforce each other and explain the reduced SA rates among care workers from lower-income non-EU countries.

Finally, it is plausible that many of the mechanisms described above, including the healthy migrant effect, the known predictors of presenteeism (e.g. job insecurity), occupational downgrading, service system illiteracy, (racial) discrimination and selection bias in recruitment may affect migrants from lower-income non-EU countries more than other migrants. Migrants from EU/EEA countries (and Switzerland) are more likely to have an EU certificate in care work and thus more job security [28], as well as better access to social security in Finland. As migrants, however, they may still be exposed to demanding social and emotional situations at work, such as language issues, which may explain the higher SA rates among migrants from Western Europe and the Global North than among their Finnish-born peers.

### Strengths and limitations

This is the first study to use data linkage methodology to estimate SA rates among migrant and non-migrant care workers. The high-quality data set covered an exceptionally large, representative sample of care workers. However, it also has some limitations. We were only able to observe SA of more than 10 calendar days. In addition, we had no information on the care workers’ health status, health behaviours, job contracts, experiences of support at work, citizenship or residence permit status or their length of residence in Finland, all of which are likely to be important predictors of SA and presenteeism.

Oversampling of men and migrants in the care sector would have allowed us to perform more detailed analyses of gender and countries of origin. As we selected a random sample of the working-age population in two consecutive cohorts, some individuals may have been included in both cohorts. The statistical analyses could not be adjusted to accommodate this factor, as it was not possible to identify these individuals. It should also be noted that we identified the occupation of each individual at the start of the follow-up but were unable to trace any occupational changes. Unemployment was censored annually if the participant was unemployed for at least two months per year. However, other events may have influenced the results, although in Finland, permanent residents aged 16–67 can claim sickness allowances in various situations (e.g. as unemployed, students, family caregivers, etc.).

The study was further limited by the lack of information on the number of people who emigrated from Finland without informing the authorities during the follow-up. This may have resulted in over-coverage of the utilised registers and an underestimation of the SA rates of migrant workers. However, over-coverage cannot explain the increased SA rates among the migrants from Western Europe and the Global North. Moreover, all participants had income from Finland during the follow-up, indicating employment or residence in Finland. These observations, the sensitivity analyses we conducted and the similar results from other studies with different designs [13,16] strengthen our confidence in the validity of our results.

## Conclusions

In this register-based follow-up study, care workers from lower-income non-EU countries had lower SA rates than Finnish-born care workers. The main theoretical explanations for this are better health, that is, migrants from lower-income non-EU countries are healthier than Finnish-born care workers, and more sickness presenteeism, that is, migrants from lower-income non-EU countries underuse their right to sickness allowance. These explanations are not mutually exclusive; they can also reinforce each other. It is likely that most of these mechanisms affect migrants from lower-income non-EU countries more than other migrants. Further research is required to test these theories.

## Supplemental Material

sj-odt-1-sjp-10.1177_14034948231168434 – Supplemental material for Sickness absence among migrant and non-migrant care workers in Finland: A register-based follow-up study Supplemental material, sj-odt-1-sjp-10.1177_14034948231168434 for Sickness absence among migrant and non-migrant care workers in Finland: A register-based follow-up study by Antero Olakivi, Anne Kouvonen, Aki Koskinen, Laura Kemppainen, Lauri Kokkinen and Ari Väänänen in Scandinavian Journal of Public Health
