# Supplementary material for: Sickness absence among migrant and non-migrant care workers in Finland: A register-based follow-up study
Source: Scand J Public Health. 2023 Apr 22;52(5):539–46. doi: 10.1177/14034948231168434 (PMC11292965; doi:10.1177/14034948231168434)
Supplement: Supplementary file 1 [file sj-odt-1-sjp-10.1177_14034948231168434.odt]

Sickness absence among migrant and non-migrant care workers in Finland: a register-based follow-up study

Supplemental material

| TABLE S1. Poisson regression rate ratios (RRs) and 95% confidence intervals (CIs) for sickness absence among care workers from different countries/regions of birth. Cohort 2011–2013. |
| --- |
|  |  | Model 1 | Model 2 |
|  | n | RR (95% CI) | RR (95% CI) |
| Finland | 37,470 |  |  |
| Western Europe and the Global North | 494 | 1.18 (1.01–1.37) | 1.16 (1.00–1.35) |
| Post-2004 EU countries | 206 | 1.00 (0.79–1.26) | 1.02 (0.81–1.30) |
| Russia, the Former Soviet Union, and the Balkan states | 496 | 0.62 (0.52–0.75) | 0.63 (0.53–0.76) |
| Global South and East | 507 | 0.71 (0.59–0.86) | 0.72 (0.60–0.87) |

Model 1: Adjusted for age (continuous) and gender.

Model 2: Adjusted for age (continuous), gender, region, income (continuous) and occupation.

| TABLE S2. Poisson regression rate ratios (RRs) and 95% confidence intervals (CIs) for sickness absence among care workers from different countries/regions of birth. Cohort 2014–2016. |
| --- |
|  |  | Model 1 | Model 2 |
|  | n | RR (95% CI) | RR (95% CI) |
| Finland | 37,185 |  |  |
| Western Europe and the Global North | 527 | 1.22 (1.06–1.41) | 1.21 (1.05–1.40) |
| Post-2004 EU countries | 298 | 0.74 (0.60–0.92) | 0.78 (0.62–0.96) |
| Russia, the Former Soviet Union, and the Balkan states | 593 | 0.68 (0.58–0.80) | 0.70 (0.60–0.83) |
| Global South and East | 700 | 0.62 (0.53–0.74) | 0.63 (0.53–0.75) |

Model 1: Adjusted for age (continuous) and gender.

Model 2: Adjusted for age (continuous), gender, region, income (continuous) and occupation.

| TABLE S3. Poisson regression rate ratios (RRs) and 95% confidence intervals (CIs) for sickness absence among care workers from different countries/regions of birth. 31–50-year-old subjects. |
| --- |
|  |  | Model 1 | Model 2 |
|  | n | RR (95% CI) | RR (95% CI) |
| Finland | 32,348 |  |  |
| Western Europe and the Global North | 645 | 1.20 (1.06–1.36) | 1.18 (1.04–1.34) |
| Post-2004 EU countries | 259 | 0.78 (0.63–0.98) | 0.79 (0.64–0.99) |
| Russia, the Former Soviet Union, and the Balkan states | 576 | 0.58 (0.49–0.69) | 0.59 (0.50–0.70) |
| Global South and East | 580 | 0.62 (0.52–0.73) | 0.61 (0.52–0.73) |

Model 1: Adjusted for age (continuous), gender and cohort.

Model 2: Adjusted for age (continuous), gender, cohort, region, income (continuous) and occupation.

| TABLE S4. Poisson regression rate ratios (RRs) and 95% confidence intervals (CIs) for sickness absence among care workers from different countries/regions of birth. Women. |
| --- |
|  |  | Model 1 | Model 2 |
|  | n | RR (95% CI) | RR (95% CI) |
| Finland | ´67,729 |  |  |
| Western Europe and the Global North | 837 | 1.21 (1.09–1.36) | 1.21 (1.08–1.35) |
| Post-2004 EU countries | 477 | 0.84 (0.72–0.99) | 0.87 (0.74–1.03) |
| Russia, the Former Soviet Union, and the Balkan states | 1,028 | 0.64 (0.57–0.73) | 0.66 (0.58–0.75) |
| Global South and East | 933 | 0.63 (0.55–0.72) | 0.64 (0.55–0.73) |

Model 1: Adjusted for age (continuous) and cohort.

Model 2: Adjusted for age (continuous), cohort, region, income (continuous) and occupation.

| TABLE S5. Poisson regression rate ratios (RRs) and 95% confidence intervals (CIs) for sickness absence among care workers from different countries/regions of birth. The middle and the highest income tertile. |
| --- |
|  |  | Model 1 | Model 2 |
|  | n | RR (95% CI) | RR (95% CI) |
| Finland | 50,028 |  |  |
| Western Europe and the Global North | 650 | 1.19 (1.06–1.35) | 1.19 (1.05–1.34) |
| Post-2004 EU countries | 319 | 0.88 (0.73–1.05) | 0.91 (0.75–1.09) |
| Russia, the Former Soviet Union, and the Balkan states | 678 | 0.59 (0.51–0.68) | 0.60 (0.52–0.69) |
| Global South and East | 689 | 0.58 (0.50–0.68) | 0.60 (0.51–0.70) |

Model 1: Adjusted for age (continuous), gender and cohort.

Model 2: Adjusted for age (continuous), gender, cohort, region, income (continuous) and occupation.
